# Successful Non-Surgical Root Canal Treatment on Auto-Transplanted Maxillary Premolar with Apical Periodontitis

**DOI:** 10.1155/2023/9389760

**Published:** 2023-06-03

**Authors:** Christopher M. Naglieri, Ellie Bash, Glen A. Karunanayake, Carlos H. R. Camargo, Takashi Komabayashi

**Affiliations:** ^1^Division of Comprehensive Oral Health - Endodontics, University of North Carolina at Chapel Hill, Adams School of Dentistry, Chapel Hill, NC, USA; ^2^Division of Comprehensive Oral Health - Periodontics, University of North Carolina at Chapel Hill, Adams School of Dentistry, Chapel Hill, NC, USA

## Abstract

Auto-transplantation is a procedure that replaces traumatized or congenitally missing teeth. While most auto-transplanted teeth are successfully integrated into recipient sites, the donor tooth may develop apical periodontitis, causing early failure. In the present case report, the periodontic resident performed the procedure on a 15-year-old male by selecting donor teeth #4 and #13 and transplanting them at recipient sites #29 and #20, respectively. After 6 weeks, the patient was referred to the endodontic resident for evaluation of tooth #20 due to symptom development. While one auto-transplanted tooth (donor tooth #4, recipient site #29) was successfully integrated, the other (donor tooth #13, recipient site #20) was unsuccessful: the patient was diagnosed with pulp necrosis and a chronic apical abscess. Because of the patient's age, collaboration among periodontic, endodontic, and orthodontic residents/specialists informed the clinical decision to pursue non-surgical root canal treatment (NSRCT) rather than extraction. The canal was cleaned and shaped to a size #80 using copious irrigation of 2.5% sodium hypochlorite (NaOCl), followed by 17% ethylenediaminetetraacetic acid (EDTA) via the EndoVac Negative Pressure Irrigation system. The tooth was dried with paper points, and then calcium hydroxide was mixed with 2.5% NaOCl and placed with an amalgam carrier 2 mm from the radiographic apex. The tooth was next temporized with Teflon tape and Fuji TRIAGE. Four weeks later, after confirming the patient was asymptomatic and tooth mobility had decreased, the canal was obturated using EndoSequence Bioceramic Root Repair Material Fast Set Putty in 2 mm incremental layers to achieve a three-dimensional fill and create an apical plug to prevent gutta-percha extrusion, then backfilled in incremental layers of gutta-percha to the cementoenamel junction (CEJ). At the 8-month follow-up, the patient was asymptomatic, and the periodontal ligament (PDL) had no signs of periapical pathology. When teeth undergoing auto-transplantation procedures develop apical periodontitis, NSRCT can be implemented.

## 1. Introduction

Auto-transplantation is an extraction of a donor's tooth from its original erupted or impacted site to a prepared recipient site or extraction socket in the same individual [1]. In young patients who experience trauma or present with congenitally missing teeth, replacement therapy may be limited due to alveolar growth development. Therefore, in most young patients, implant-retained restorations or fixed partial dentures are only an appropriate treatment plan in the later stages of development. For this reason, auto-transplantation can successfully replace congenitally missing teeth and teeth lost to trauma or caries in young adolescent patients [2]. Auto-transplanted teeth can also induce bone growth, which will continue as the patient matures and develops [3]. When the procedure is performed correctly, success rates of revascularization are approximately 85%, and patients can expect a long-term tooth survival rate of 83–98% [2, 4, 5]. However, not all cases of auto-transplantation are successful. When a donor's tooth is implanted with periodontal ligament (PDL) damage, there is a risk of ankylosis with replacement resorption or pulpal necrosis with apical periodontitis [1]. The patient in this case report presented with congenitally missing mandibular second premolar; this condition has a prevalence of 2.5–4% [6]. This case report addresses how to manage treatment complications with auto-transplantation, including apical periodontitis, marginal bone loss, and unfavorable mobility, which clinicians may consider a failure and ultimately lead to tooth extraction. With the successful management of pulp necrosis and apical periodontitis after auto-transplantation by non-surgical root canal treatment (NSRCT), adverse complications can be overcome and lead to a favorable outcome.

## 2. Case Report

**Figure 1:**
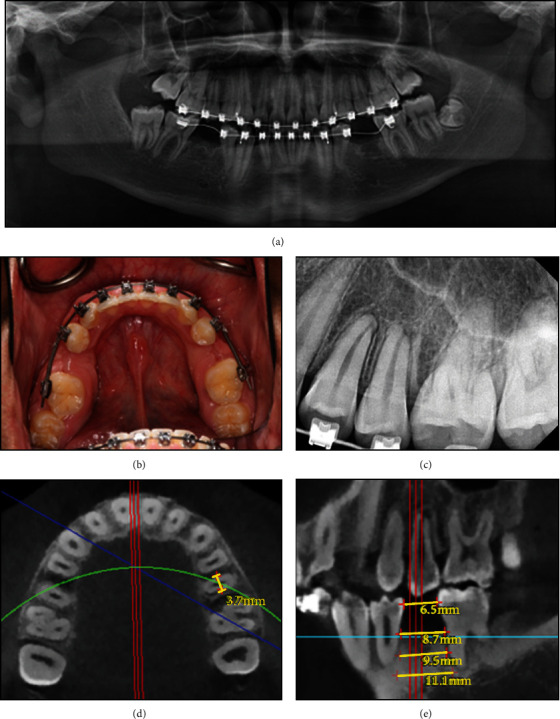
Pre-operative records of auto-transplantation. (a) Panoramic radiograph showing congenitally missing teeth #20 and #29. (b) Pre-operative periapical radiograph of tooth #13. (c) Clinical photo of mandibular arch showing congenitally missing teeth #20 and #29. (d) Pre-operative CBCT of the apical third of the root of tooth #13 (donor tooth). (e) Pre-operative CBCT of recipient site for transplanting tooth #20.

The 15-year-old male patient had undergone auto-transplantation therapy at the University of North Carolina at Chapel Hill (UNC-CH) Adams School of Dentistry (ASOD) Graduate Periodontic Clinic. The treatment planning included a full periodontal examination, such as probing depths and plaque index. Cone beam computed tomography (Orthophos SL 3D, Dentsply Sirona, Charlotte, NC) was used to evaluate recipient sites and donor teeth for surgical planning. The periodontic resident determined the auto-transplantation procedure by selecting donor teeth #4 and #13 and transplanting the teeth at recipient sites #29 and #20, respectively (Figures 1(a), 1(b), 1(c), 1(d), and 1(e)).

**Figure 2:**
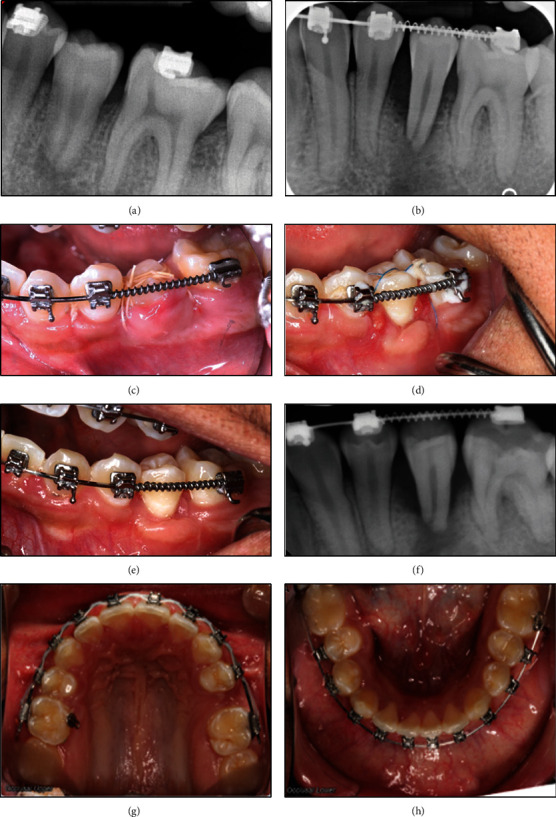
Post-operative records of auto-transplantation. (a) Post-operative periapical radiograph of immediate auto-transplantation of tooth #13 (donor tooth) to the socket of tooth #20 (recipient site). (b) Periapical radiograph 1-week follow-up of auto-transplantation of tooth #20. (c) Clinical photo 1-week post-operative of auto-transplantation of tooth #20. (d) Clinical photo 3-week post-operative of auto-transplantation of tooth #20. (e) Clinical photo 6-week post-operative of auto-transplantation of tooth #20. (f) Periapical radiograph showing 6-week post-operative follow-up of auto-transplantation of tooth #20. (g) Clinical photo 6-week post-operative of auto-transplantation: maxillary occlusal view. Teeth #4 and #13 were donor teeth, and spaces were closing. (h) Clinical photo 6-week post-operative of auto-transplantation: mandibular occlusal view. Teeth #20 and #29 were recipient sites, and spaces were closed.

All auto-transplantation procedures were performed by the periodontic resident at the UNC-CH ASOD Graduate Periodontic Clinic. While one auto-transplanted tooth (donor tooth #4, recipient site #29) was successfully integrated into recipient sites, the other auto-transplanted tooth (donor tooth #13, recipient site #20) developed severe bone loss and apical periodontitis. Therefore, after 6 weeks, the patient was referred to UNC-CH ASOD Graduate Endodontic Clinic for evaluation of #20 (Figures 2(a), 2(b), 2(c), 2(d), 2(e), 2(f), 2(g), and 2(h)).

Upon evaluation at UNC-CH ASOD Graduate Endodontic Clinic, the patient's chief complaint was minor pain when biting and the presence of a sinus tract on the mandibular left quadrant. His medical history was unremarkable. He had no drug allergies, medications, or cardiac/joint diseases requiring antibiotic prophylaxis. His dental history included routine dental care, low caries risk, and good oral hygiene. The extra oral exam and perioral soft tissue exam were within normal limits. His temporomandibular joint function was normal, without any deviation upon opening or discomfort upon palpation. The intraoral examination revealed normal soft tissues with neither pathology nor symptoms. Clinically, tooth #20 had no restorative or carious defects. Examination did not reveal any cracks or fractures, and discomfort upon percussion, palpation, and bite stick testing was minor. Teeth #19 and #21 were tested with EndoIce (Coltène Whaledent, Cuyahoga Falls, OH, USA) and responded within normal limits. Tooth #20 did not respond to EndoIce. The radiographic examination revealed normal PDL space around teeth #19 and #21 but widening of the PDL space, loss of lamina dura, and localized loss of marginal bone on the mesial and distal of tooth #20 (Figure 2(f)). The endodontic diagnosis for this patient was pulp necrosis with chronic apical abscess.

Treatment options discussed with the patient included NSRCT or extraction. After considering the risks and benefits of both procedures, an agreement was made to proceed with NSRCT due to recent auto-transplantation. The NSRCT for this tooth started the day of the evaluation to alleviate the patient's discomfort and sinus tract. Local infiltration of 3.6 ml of 4% articaine with 3.6 ml of 1 : 100,000 epinephrine (Septocaine; Septodont, New Castle, DE, USA) was administered. Dental clamp and dental dam modifications were necessary to achieve the appropriate isolation due to the present orthodontic bracket and archwire; adjunctive endodontic isolation was performed with OraSeal (Ultradent Products, South Jordan, UT, USA). Access to cavity preparation was challenging due to multiple factors, including tooth mobility and maxillary premolar anatomy in a mandibular arch. Magnification up to 20× and illumination of 125 K (Prima, Labomed, Gurgaon, India) were used for canal identification and to reduce the possibility of missed canals.

**Figure 3:**
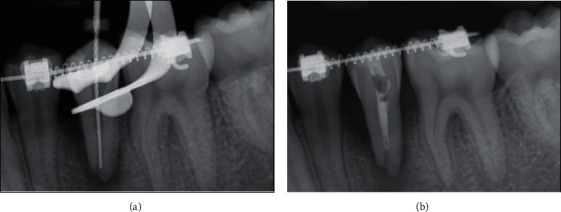
Initial endodontic treatment. (a) Periapical radiograph of working length of tooth #20. (b) Periapical radiograph of calcium hydroxide placement of tooth #20.

A single canal was found, and the working length was determined using an electronic apex locator (Root ZX II, Morita, Kyoto, Japan); measurement films were taken at multiple angulations to visualize canal configurations and apical termini. Debridement was accomplished using hand instrumentation with K-type and Hedstrom files (Dentsply-Sirona, Johnson City, TN, USA) to a size #80 (Figure 3(a)). Copious irrigation of 2.5% sodium hypochlorite (NaOCl) (Henry Schein, Melville, NY, USA) was used during the cleaning and shaping process, followed by a final rinse of 17% ethylenediaminetetraacetic acid (EDTA) (Henry Schein, Melville, NY, USA) to remove the smear layer. The irrigation solutions were delivered via the EndoVac Negative Pressure Irrigation system (Discus Dental, Culver City, CA, USA). The tooth was dried with medium endodontic paper points (Dentsply-Sirona, Johnson City, TN, USA) until the paper points were stiff. Calcium hydroxide (Sultan Healthcare, York, PA, USA) was mixed with 2.5% NaOCl and placed with an amalgam carrier 2 mm from the radiographic apex (Figure 3(b)). The tooth was then temporized with Teflon tape and Fuji TRIAGE (GC America, Alsip, IL, USA).

**Figure 4:**
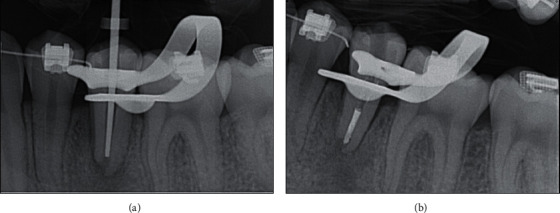
Obturation radiographs. (a) Periapical radiograph of endodontic plugger measurement of tooth #20. (b) Periapical radiograph of 4 mm apical plug of tooth #20.

The patient presented 4 weeks later for follow-up and obturation. No symptoms were observed, the chief complaint had been resolved, and mobility had decreased to class two. Administration of local anesthesia and a rubber dam placement technique was used. A nickel–titanium plugger (SybronEndo, Kerr, Brea, CA, USA) was selected to obtain an appropriate apical fit of 2 mm from the radiographic apex and working length (Figure 4(a)). After the nickel–titanium plugger placement was confirmed, the canals were dried with medium paper points (Dentsply-Sirona, Johnson City, TN, USA). Once the canal was dried thoroughly, it was obturated using EndoSequence Bioceramic Root Repair Material Fast Set Putty (Brasseler, Savannah, GA, USA) in 2 mm incremental layers. A radiograph was exposed to ensure a proper 2–4 mm apical seal (Figure 4(b)). The aim of this technique was to achieve a three-dimensional fill and create an apical plug to prevent gutta-percha extrusion. After the apical plug, backfill was performed with a Kerr Backfill unit (SybronEndo, Kerr, Orange, CA, USA) in incremental layers of gutta-percha to the cementoenamel junction (CEJ).

**Figure 5:**
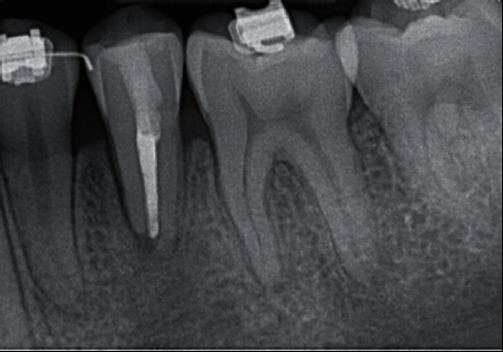
Periapical radiograph of immediate post-operative obturation of tooth #20.

When obturation was complete, excess gutta-percha was removed from the chamber using a heated posterior endodontic plugger (Kerr Dental, Orange, CA, USA). The tooth was restored by placing 37% ortho-phosphoric acid etch on the enamel for 30 seconds and rinsing with water. Once the tooth was dry, Optibond Solo Plus (Kerr Dental, Orange, CA, USA) was coated and scrubbed in the endodontic access for 20 seconds, air-dried, and cured for 30 seconds. Lastly, a packable composite (Filtek Supreme, 3M, Two Harbors, MN, USA) was placed in 2 mm incremental layers until access was filled and then cured for 1 minute. The composite was smoothed and polished with a football-shaped bur (Komet USA, Rock Hill, SC), and occlusion was checked with articulating paper until acceptable occlusal contact was determined. The final post-operative radiograph was exposed (Figure 5). The patient was referred to his orthodontist for continued care.

**Figure 6:**
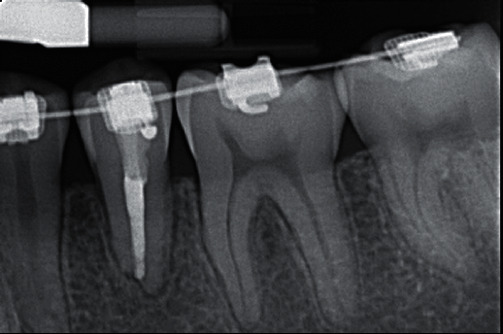
Periapical radiograph of tooth #20 at 8-month post-operative follow-up.

The patient was recalled for an 8-month follow-up to NSRCT, and the endodontic outcome was considered favorable. The radiograph showed normal PDL, and the lamina dura had no signs of periapical pathology. The patient has remained asymptomatic after the completion of NSRCT; a radiographic exam revealed complete healing of periapical pathosis (Figure 6). The permanent restoration was intact without any evidence of leakage. All probing depths were within normal limits with normal physiological mobility.

## 3. Discussion

Over years of dental implant and fixed prosthesis development, auto-transplantation has seen a slight decrease among clinicians for several reasons. The criteria for patients who qualify for auto-transplantation are numerous. Careful interpretation of three-dimensional cone beam computed tomography to assess the donor tooth, recipient site, and surrounding structures must be conducted [7]. Root development must also be considered: the highest success for re-vascularization of the donor tooth occurs when root development is one-half to two-thirds complete [1]. When compared to the Moorrees' classification, the ideal root development for auto-transplantation would be class 3 and class 4 [8]. However, in cases with complete or near-complete root development, Abella and Roig suggest NSRCT preemptively or 2 weeks after due to poor revascularization and the risk of replacement root resorption [9]. Furthermore, patient variables such as age, compliance, and orthodontic treatment may complicate selection. However, auto-transplantation still holds as an important treatment option in young patients with missing teeth due to trauma or decay as well as congenitally missing teeth unable to receive fixed solutions [10].

In successful auto-transplantation cases, the donor's tooth regains a healthy PDL for continued bone and soft tissue growth [9]. In this clinical case, one auto-transplanted tooth (donor tooth #4, recipient site #29) was successfully integrated into a recipient site. In some cases, a side effect of the donor's tooth is the presence of pulp canal obliteration; however, the tooth retains its vitality and only about 8% of cases result in true necrosis despite pulp sensibility tests. There are cases, such as the one presented, where complications result in apical periodontitis and a failing donor tooth regardless of the correct criteria for revascularization. This outcome can be due to poor handling of the extracted tooth, destroying PDL cells that lead to resorption, or contamination of the apical foramen with bacteria during or after the surgery. Bacterial colonization leads to apical periodontitis and failure of revascularization, which causes bone resorption and early tooth loss. In the present case, the initial etiology appears to have been bacterial contamination that colonized the root canal space to create bone resorption, a sinus tract, and mobility [11].

This case was successfully managed due in part to the proper disinfection of the root canal system and effective collaboration among periodontics, endodontics, and orthodontics. Upon reviewing the case, all specialties agreed that early tooth loss would pose a problem for the patient due to his young age. If the tooth was ultimately extracted, orthodontics would need to move adjacent teeth to allow proper space for a future implant. Once the patient completed craniofacial growth, the periodontist would carry out implant therapy with an implant-retained crown. Even with long-term data on implant success, implants may result in implant loss and further complications that could cause damage to the existing alveolar bone and adjacent teeth. However, even in teeth with periapical lesions, endodontic success rates are approximately 80% and ultimately could lead to successful retention of the auto-transplanted donor tooth [12].

## 4. Conclusion

This case demonstrates how adverse outcomes of auto-transplantation, such as apical periodontitis, can be remedied with NSRCT. With endodontic treatment, the body can begin healing once the etiology of bacteria is removed and the root canal system is sealed. Disinfection with sodium hypochlorite and placement of calcium hydroxide in the canal space significantly reduced bacterial colonies and biofilm, allowing localized healing of the periapical tissue. At the patient's second visit, it was evident that healing had begun with the disappearance of the sinus tract and the resolution of the patient's chief complaint. Over the next 8 months, the patient's immune system was able to promote bony deposition by osteoblasts through the adaptative immune response and the formation of new healthy PDL. With the teamwork from the periodontal, orthodontic, and endodontic residents, the patient could continue orthodontic treatment and successfully retain the auto-transplanted donor tooth. This case report indicates that NSRCT can successfully restore teeth which develop apical periodontitis during auto-transplantation.

## Data Availability

Data supporting this research article are available from the corresponding author or first author on reasonable request.
